# Electronic health records-related determinants of healthcare professionals' burnout and mitigation strategies: systematic review and meta-analysis

**DOI:** 10.3389/fpubh.2026.1751521

**Published:** 2026-03-13

**Authors:** Yi Yang, Rui Shi, Zeng Wang, Jia Xu, Jiaxi Xie, Jialin Liu

**Affiliations:** Information Center of West China Hospital, Sichuan University, Chengdu, China

**Keywords:** burnout, electronic health record, healthcare professionals, meta-analysis, mitigation strategies

## Abstract

**Background:**

While the adoption of electronic health records (EHRs) has become widespread, it has been accompanied by a concurrent exacerbation of burnout among healthcare professionals. However, existing research has predominantly focused on single professional groups, lacking comprehensive multi-group analysis and the identification of key modifiable mitigation factors.

**Methods:**

Following the PRISMA guidelines, we systematically searched for relevant literature published between 2005 and 2025. A total of 41 studies, encompassing 54,443 healthcare professionals, were included. A meta-analysis was conducted to assess the association between EHR use and occupational burnout, with subgroup analyses performed to examine differences across various professional groups and assessment tools. Sensitivity analysis was conducted to reduce the bias.

**Results:**

The use of EHRs was found to significantly associated with an increase the risk of occupational burnout (OR = 2.49, 95% CI: 1.82–3.41), which was also supported after sensitivity analysis (OR = 1.98, 95% CI: 1.40–2.80). The subgroup analysis revealed that the occurrence rate of burnout was highest in studies using other tools (39.3%), followed by those using the MBI-HSS (36.0%) and was lowest in studies employing the mini-Z (31.8%). This association was evident across multiple groups, The highest occurrence rate among physicians was 38.1%, followed by residents (37.5%), and then nurses (27.8%). The primary contributing factors were poor EHR design, excessive time spent on documentation, and heavy administrative burdens. Conversely, mitigations such as system optimization and the provision of medical scribes have been proposed as potentially beneficial approaches for alleviating burnout.

**Conclusion:**

EHR use is closely linked to occupational burnout across a broad spectrum of healthcare professionals. There is a critical need for targeted system optimization and the development of tailored mitigation strategies to reduce this growing problem.

## Introduction

With the accelerating process of digitalization in healthcare, the electronic health record (EHR) has become an indispensable tool in modern medical systems. Its value in enhancing healthcare quality, optimizing clinical workflows, and promoting information sharing has been widely recognized (1–3). However, the widespread adoption of EHRs is also accompanied by a series of potential issues, among which the exacerbation of professional burnout among healthcare personnel is a significant concern. Professional burnout not only reduces the efficiency of healthcare services and compromises patient safety but also leads to increased turnover rates among healthcare professionals, posing a severe challenge to the stability of the healthcare system (4–6).

Currently, numerous studies have explored the association between EHRs and professional burnout among healthcare personnel, but existing research has several limitations. Most studies focus on a single group of healthcare professionals (such as physicians or nurses), lacking a holistic analysis of multiple groups (including physicians, nurses, technicians, administrative staff). This makes it difficult to comprehensively reveal the commonalities and differences in how EHRs contribute to burnout across different professional roles. For example, one report including only physicians stated that spending excessive time on documentation and workflows is a primary cause of professional burnout (7). Another study on family physicians showed that approximately 25% were highly dissatisfied with the usability and satisfaction of their EHR system (8). For Advanced Practice Registered Nurses (APRNs), 50.3% strongly indicated that EHRs increased their daily frustration, contributing to professional burnout (9). On the other hand, existing research has largely been confined to phenomenological descriptions and associative analyses (10, 11), lacking a systematic identification of the modifiable key factors contributing to EHR-related burnout. Additionally, research on effective mitigation strategies for EHR-related burnout is scarce and fragmented. One study offered practical suggestions for addressing EHR-related burnout only within a Canadian healthcare organization (12), while mitigation strategies targeting professional burnout across multiple groups of healthcare personnel are particularly insufficient.

This study employs systematic review and meta-analysis to synthesize global research findings. For the first time, it incorporates multiple groups of healthcare personnel into a unified analytical framework to systematically evaluate the impact of EHRs on professional burnout, clarify the strength of this association, analyze differences across various job roles and identify key risk factors. The findings will provide evidence-based insights for EHR system optimization and for healthcare institutions in developing mitigation strategies for professional burnout, offering a scientific basis for optimizing EHR applications and alleviating burnout among healthcare professionals.

## Methods

### Protocol and registration

This systematic review was conducted in accordance with the PRISMA (Preferred Reporting Items for Systematic Reviews and Meta-Analyses) guidelines.

### Definition of burnout

In this study, the definition of professional burnout is primarily based on the Maslach Burnout Inventory-Human Services Survey (MBI-HSS) (10, 13). This instrument assesses burnout across three dimensions: emotional exhaustion, depersonalization, and reduced personal accomplishment. Using the definition of burnout from the original study. The specific criteria for high levels of burnout in each dimension were defined as follows: a score of ≥27 for high emotional exhaustion, a score of >10 for high depersonalization, and a score of < 33 for low personal accomplishment. Additionally, some studies in our review defined burnout using alternative instruments, such as the Stanford Physician Well-being Survey (14) or the mini-Z. We categorized the included studies based on the measurement tool and the specific definition of burnout they employed.

### Search strategy

A systematic literature search was conducted in PubMed, Embase, and Web of Science for relevant English-language articles published between January 1, 2005, and July 31, 2025. To retrieve literature on EHR systems, the following search terms were used: “electronic health record,” “EHR,” “EMR,” “computerized physician order entry,” “CPOE,” “clinical decision support system,” and “CDSS.” For the concept of burnout, the terms “burnout,” “burn-out,” “alert fatigue,” and “exhaustion” were employed. To define our study population, a range of healthcare professionals was considered, including “physicians,” “doctors,” “medical staff,” “nurses,” “clinicians,” “medical student,” and “healthcare professional.” These terms were combined using Boolean logic. The detailed search strategy is available in Supplementary Table 1.

### Inclusion and exclusion criteria

#### Inclusion criteria

1. Studies that assessed EHR-related burnout using MBI-HSS, mini-Z, or other self-report measures.
2. Studies that examine the use of general EHR systems or specific supportive systems, such as CPOE.
3. Studies that evaluated burnout among healthcare professionals and their individual psychological responses to EHR systems.

#### Exclusion criteria

1. Duplicate publications.
2. Literature not relevant to EHRs or occupational burnout.
3. Non-original research articles, including qualitative studies, editorials, commentaries, conference abstracts, and letters.
4. Studies with an unclear description of the methodology or an unclear definition of EHR-related outcomes.

### Data extraction and synthesis

To ensure the integrity and reliability of the data, enhance the efficiency of data extraction, and minimize subjective bias, two reviewers with different professional backgrounds independently performed the study selection (inclusion and exclusion process), quality assessment, and data extraction. The following data were extracted from the eligible studies: first author, publication year, country, study design, total sample size, and study outcomes. The primary outcome was whether professional burnout occurred among physicians, nurses, or residents as reported in cross-sectional studies.

### Risk of bias assessment

Two reviewers assessed the completeness, verifiability, and quality of the included studies using the Joanna Briggs Institute (JBI) checklist (15) (Supplementary Table 2) and the Newcastle-Ottawa Scale (NOS) (16).

### Statistical analysis

Literature screening was performed using EndNote X9 software, and meta-analysis was conducted using Review Manager 5.4 software. Heterogeneity was assessed using the *I*^2^ statistic, with statistical significance set at *P* < 0.05. If no significant statistical heterogeneity was present (*I*^2^ < 50%), a fixed-effect model was used to pool the results; otherwise (*I*^2^ ≥ 50%), a random-effects model was employed (16). Continuous variables were summarized using the mean and standardized mean difference (SMD), while rates were extracted for categorical variables. For cross-sectional studies, the effect size measure was the OR value for burnout and its corresponding 95% confidence interval (CI). We further conducted subgroup analyses based on the burnout assessment tools and different groups of healthcare professionals. Publication bias was assessed using a funnel plot. After excluding outliers, small studies, and high risk-of-bias studies, sensitivity analysis was conducted to reduce the bias.

## Results

### Literature search and study selection

**Figure 1:**
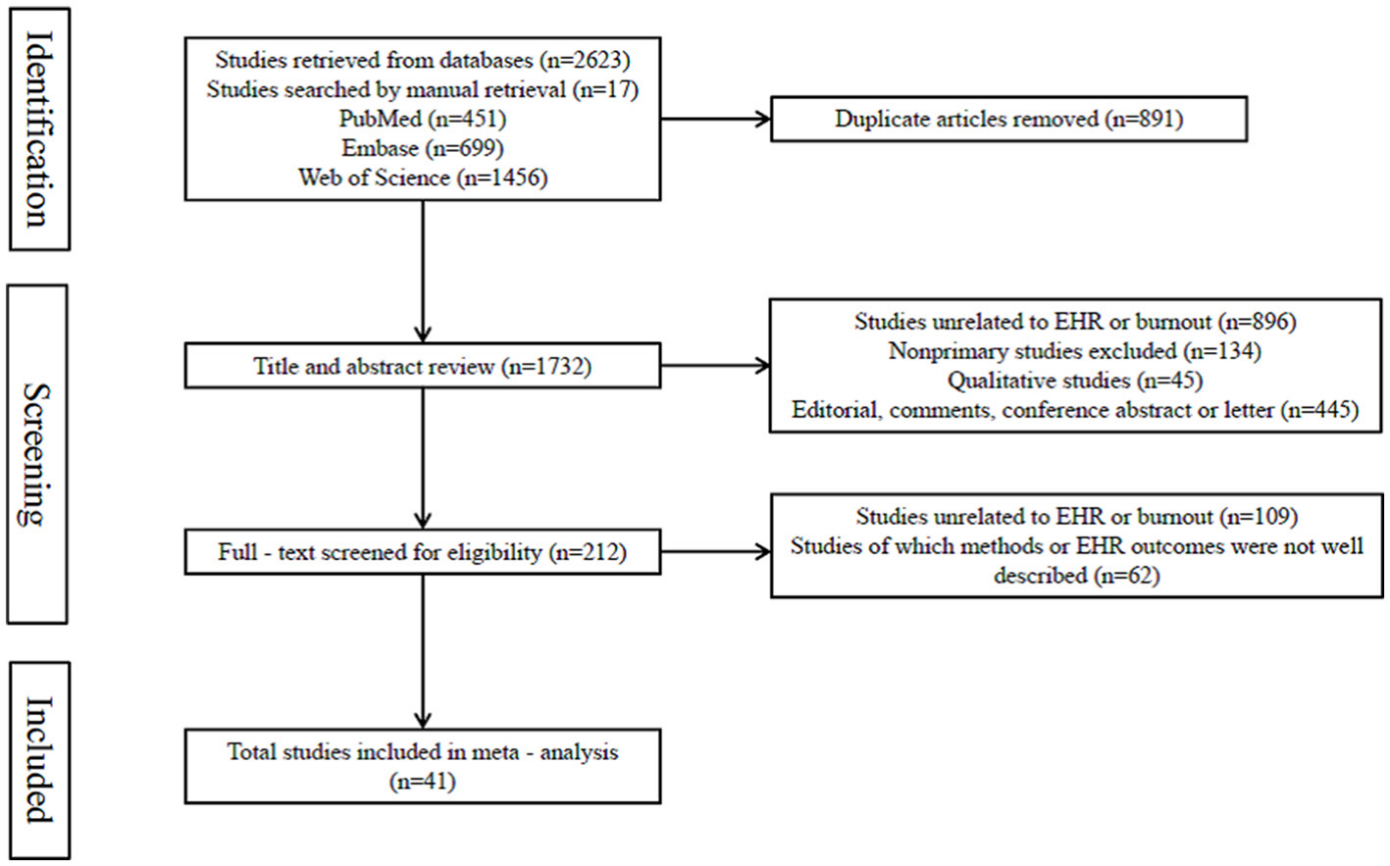
Study selection process.

A total of 2,640 articles were identified through a combination of database searches and manual retrieval. The database search was conducted across PubMed, Embase, and Web of Science, supplemented by 17 articles found through manual searching. After an initial screening of titles and abstracts, 1,520 articles were excluded for the following reasons: 896 were unrelated to EHRs or professional burnout; 134 were not original research articles; 45 were qualitative studies; and 445 were editorials, conference abstracts, or letters. The remaining 212 articles underwent full-text review, which led to the further exclusion of 171 articles: 109 were found to be unrelated to EHRs or professional burnout upon full-text assessment, and 62 had unclear methodology or an undefined EHR-related outcome. Ultimately, 41 studies met the inclusion criteria and were included in the meta-analysis for the subsequent evaluation of the impact of EHRs on professional burnout among various groups of healthcare professionals (Figure 1).

### Characteristics of the included studies

The final analysis included 41 studies, published between 2005 and 2025, conducted across regions in Canada, the United States, Iran, and Saudi Arabia. These studies collectively comprised 54,443 healthcare professionals. The sample sizes of the included studies varied considerably, ranging from 40 to 15,505 participants, with response rates ranging from 3.3% to 71.13%. The most used assessment tool for burnout was the MBI-HSS, which was utilized in 19 of the 41 studies (46.3%). Additionally, 11 studies (26.8%) employed the mini-Z burnout assessment (Table 1).

**Table 1:** Characteristics of cross-sectional studies.

**Author**	**Data collection**	**Region**	**Participants**	**Sample (total)**	**Burnout cases**	**Burnout occurrence (%)**	**Measurement**	**Response rate (%)**
Shanafelt et al. (25)	2016	United States	Physicians and other clinician staff	6,560	3,586	54.66	MBI	19.2
Tawfik et al. (34)	2014	United States	Physicians	1,934	517	26.73	MBI	70
Tawfik et al. (35)	2015	United States	Physicians and other clinician staff	1,5505	5,065	32.67	MBI	70.4
Kroth et al. (36)	2015	United States	Physicians	41	5	12.00	Other	Not reported
Kutney-Lee et al. (30)	2021	United States	Nurses	1,2004	3,160	26.30	MBI	Not reported
Olson et al. (37)	2016	United States	Physicians	557	267	47.94	MBI	44
Tai-Seale et al. (38)	2016	United States	Physicians	919	331	36.02	mini-Z	71.13
Apaydin et al. (26)	2016	United States	Physicians and other clinician staff	116	62	53.45	MBI	Not reported
Livaudais et al. (11)	2016	United States	Physicians and other clinician staff	281	127	45.20	Other	44
Tran et al. (39)	2017	United States	Physicians and other clinician staff	107	41	38.32	mini-Z	56
Marckini et al. (40)	2017	Canada and United States	Physicians	110	44	40.00	MBI	28.7
Gardner et al. (41)	2017	United States	Physicians	1792	465	25.95	mini-Z	42.7
Hilliard et al. (42)	2017	United States	Physicians and other clinician staff	422	116	27.49	mini-Z	39.3
Higgins et al. (43)	2017	United States	Residents	230	86	37.39	Other	Not reported
Czernik et al. (28)	2017	United States	Residents	84	30	35.71	Other	67
Melnick et al. (10)	2017	United States	Nurses	1,282	539	42.00	MBI	9.9
Domaney et al. (44)	2017	United States	Psychiatry Residents and Faculty	40	25	62.50	MBI	Not reported
Hauer et al. (45)	2018	United States	Physicians	1,165	624	53.56	mini-Z	8.86
Gajra et al. (46)	2018	United States	Physicians	163	109	67.00	Other	Not reported
Adler-Milstein et al. (47)	2018	United States	Physicians	122	44	36.07	MBI	37
Somerson et al. (27)	2018	United States	Residents	203	78	38.42	MBI	Not reported
Melnick et al. (48)	2018	United States	Physicians	870	397	45.63	MBI	69.6
Coleman et al. (49)	2018	United States	Physicians	872	360	41.28	MBI	34.3
Abraham et al. (50)	2018	United States	Nurses	396	100	25.25	mini-Z	Not reported
Kondrich et al. (51)	2018	Canada and United States	Physicians	416	206	49.52	MBI	59.4
Kroth et al. (29)	2019	United States	Physicians and other clinician staff	282	127	45.04	Other	44.1
Tajirian et al. (4)	2019	Canada	Physicians and trainee	208	51	24.52	mini-Z	43.8
Mandeville et al. (52)	2019	United States	Physicians and other clinician staff	2,468	539	21.84	mini-Z	39.5
Tiwari et al. (53)	2019	United States	Physicians and other medical staff	128	65	50.8	MBI	Not reported
Sinha et al. (54)	2019	United States	Physicians	856	276	32.24	Other	73
Anderson et al. (55)	2019	United States	Physicians and trainee	756	373	49.34	MBI	9.2
McPeek-Hinz et al. (56)	2019	United States	Physicians	1,310	681	52.00	MBI	3.3
Nair et al. (57)	2019	United States	Physicians	457	106	23.19	MBI	Not reported
Jha et al. (58)	2020	United States	Physicians and other medical staff	100	52	52.00	Other	55.9
Esmaeilzadeh and Mirzaei (59)	2020	Iran	Physicians and other medical staff	368	134	36.41	Other	Not reported
Holzer et al. (60)	2020	United States	Physicians and trainee	222	84	37.84	Other	16.2
Baxter et al. (61)	2020	United States	Physicians	609	307	50.40	mini-Z	60.4
Wilkie et al. (62)	2021	Canada	Physicians	103	41	39.80	MBI	40.9
Almulhem et al. (63)	2021	Saudi Arabia	Physician trainees	182	73	40.10	mini-Z	Not reported
Lou et al. (64)	2021	United States	Physician trainees	75	32	42.70	Other	Not reported
Tajirian et al. (65)	2023	Canada	Physicians	128	20	15.60	mini-Z	50

### Meta-analysis of included studies

**Figure 2:**
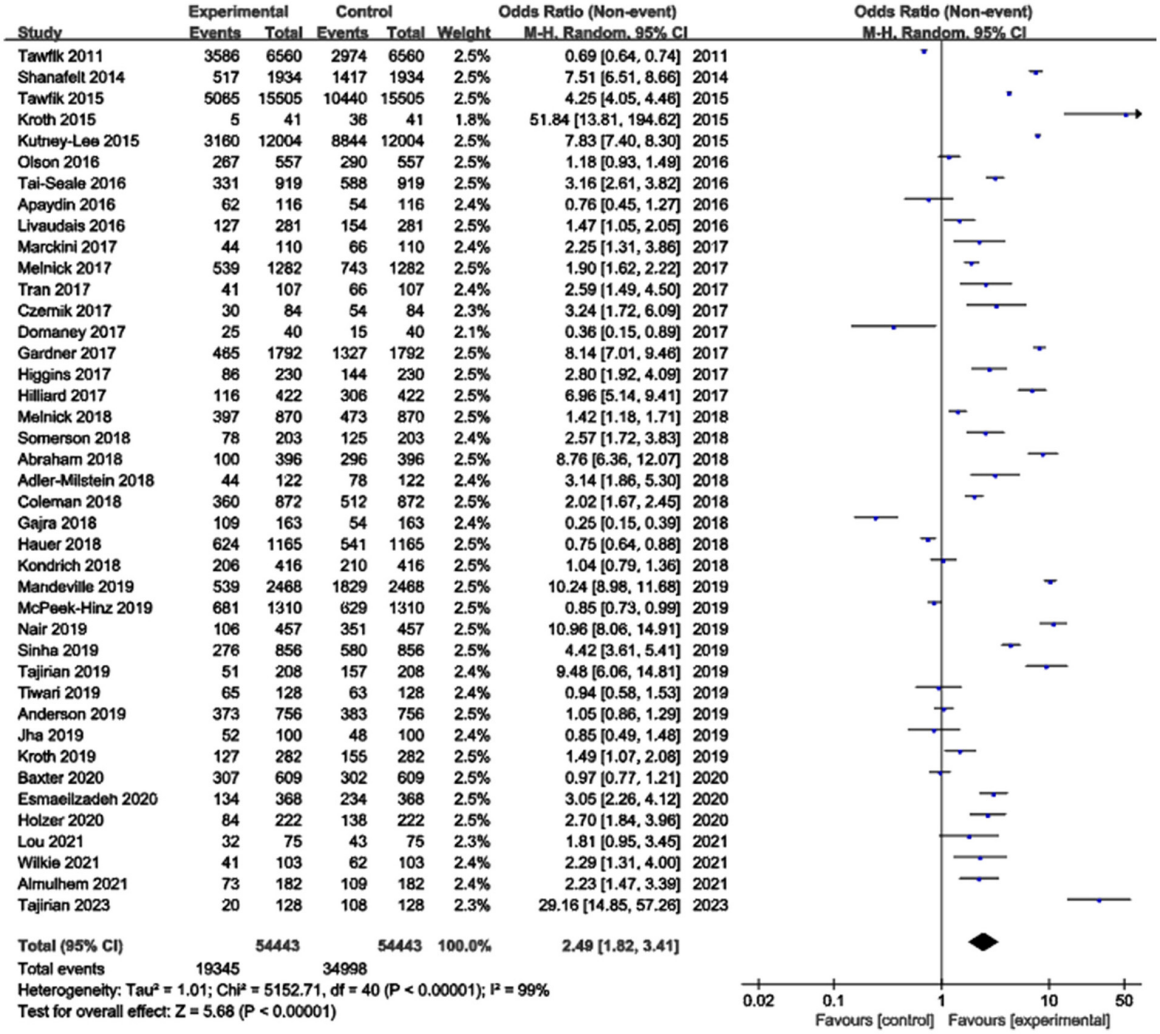
Meta-analysis of the association between EHR use and professional burnout.

**Figure 3:**
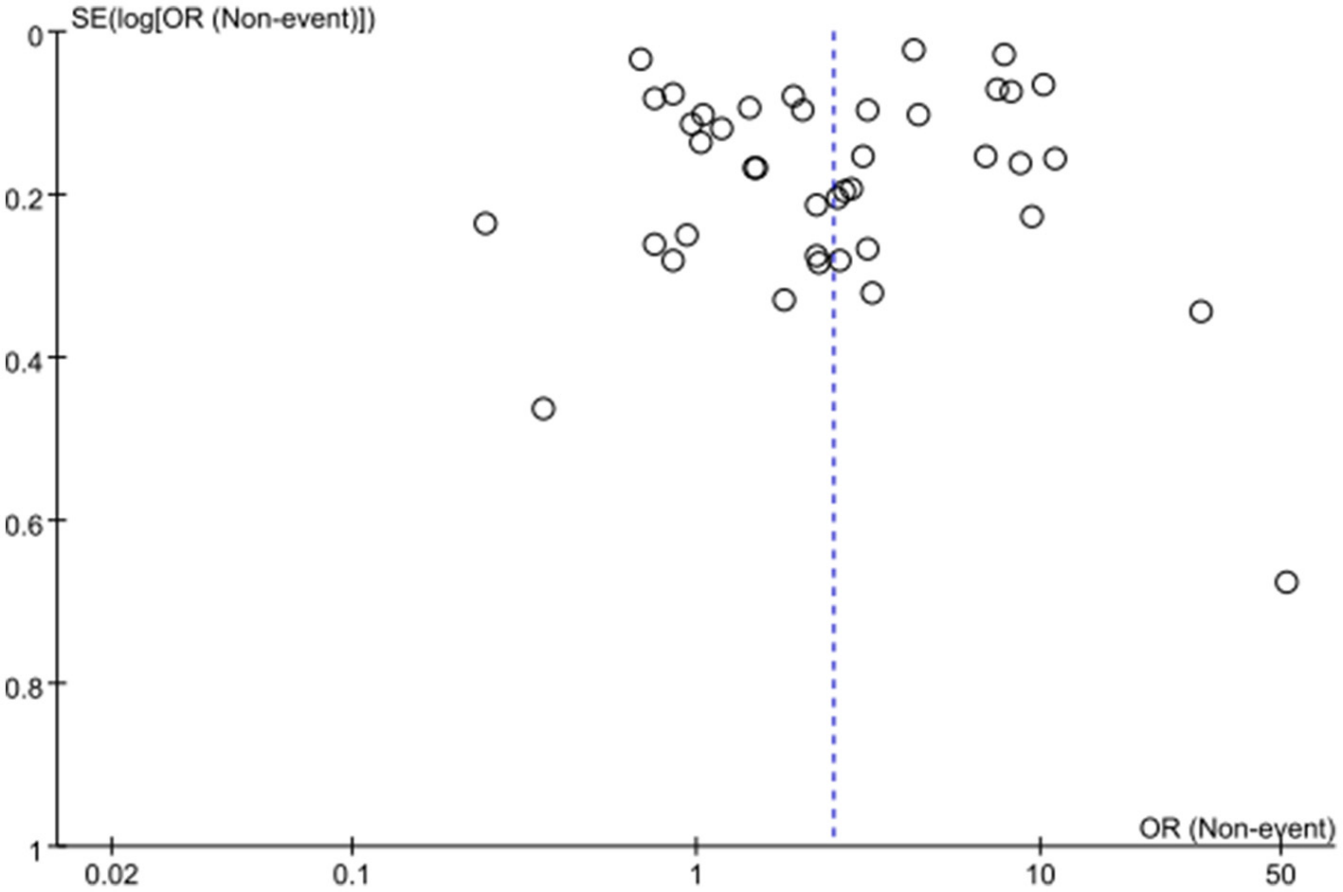
Funnel plot for publication bias.

The meta-analysis examining the association between EHR use and burnout risk included 41 studies with a total of 54,443 healthcare professionals. The heterogeneity test indicated substantial heterogeneity among the studies (*I*^2^ = 99%), leading to the application of a random-effects model. The results demonstrated that EHR use was significantly associated with an increased risk of professional burnout, with a pooled Odds Ratio (OR) of 2.49 (95% CI: 1.82–3.41, *p* < 0.00001) (Figure 2). Publication bias was assessed using a funnel plot, which revealed no significant publication bias. The points in the funnel plot were symmetrically distributed, and there was no statistically significant evidence of publication bias (Figure 3). Detailed results of the quality evaluation are presented in Supplementary Table 3.

### Subgroup analysis based on burnout assessment tools

**Figure 4:**
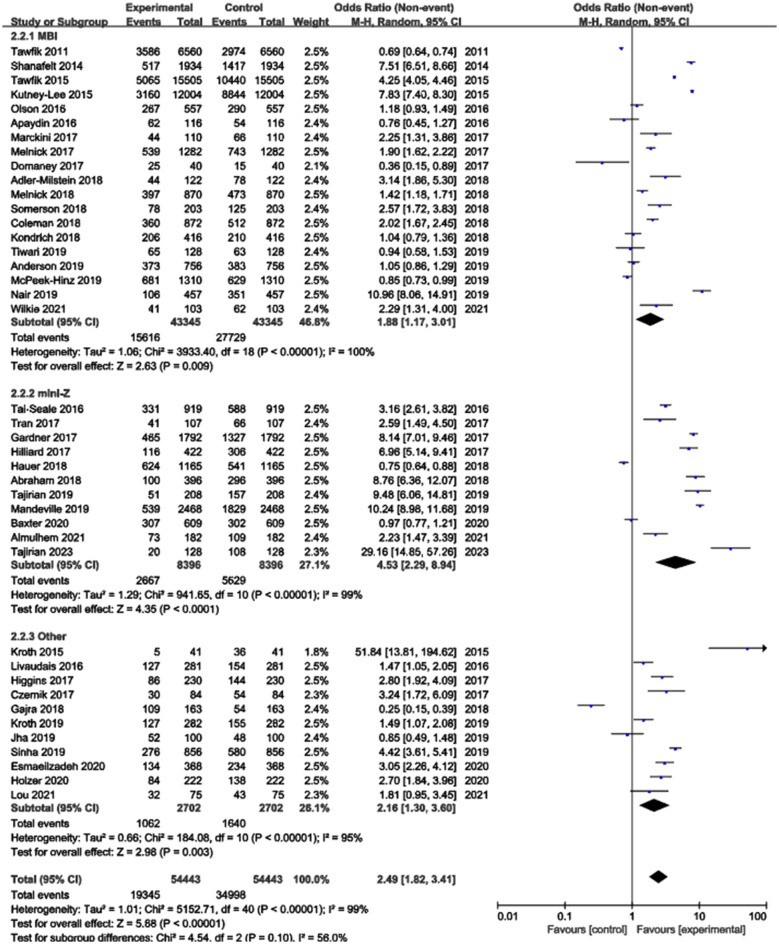
Subgroup analysis based on burnout calculation methods.

**Figure 5:**
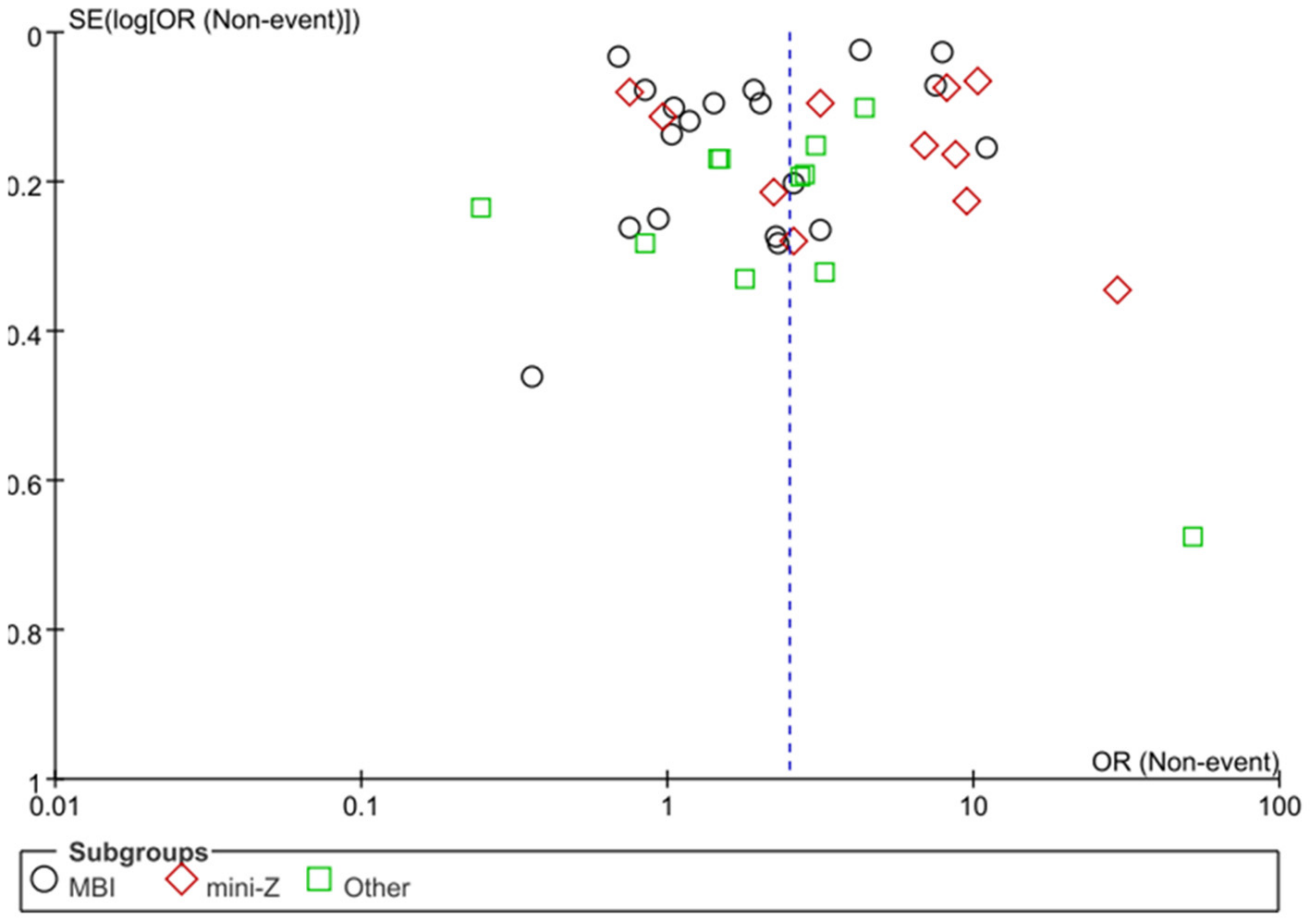
Funnel plot for publication bias.

Three distinct burnout assessment tools were identified among the included studies: the MBI-HSS, the mini-Z, and other instruments. Due to the presence of significant heterogeneity within each subgroup (*I*^2^ > 50%), a random-effects model was applied for all analyses. The subgroup analysis revealed that the occurrence rate of burnout was highest in studies using other tools (39.3%), followed by those using the MBI-HSS (36.0%) and was lowest in studies employing the mini-Z (31.8%) (Figure 4). However, these differences in occurrence rate among the three assessment methods were not statistically significant (*p* = 0.10). Publication bias was assessed using funnel plots, which showed a symmetrical distribution of points, suggesting no significant publication bias (Figure 5).

### Subgroup analysis based on healthcare professional populations

**Figure 6:**
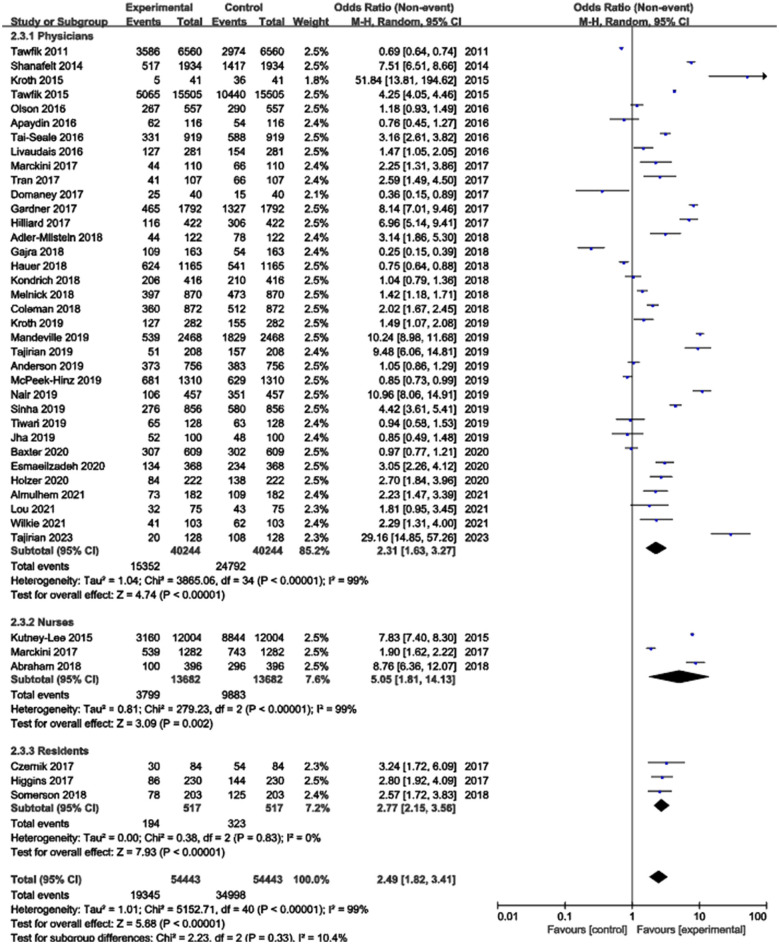
Subgroup analysis based on healthcare professional populations.

The included studies were categorized into three primary groups based on the study population: physicians, nurses, and residents. Given the significant heterogeneity observed within each of these subgroups (*I*^2^ > 50%), a random-effects model was utilized for all meta-analyses. The subgroup analysis confirmed a significant association between EHR use and professional burnout across all three populations. Specifically, the pooled OR was 2.31 (95% CI: 1.63–3.27, *p* < 0.00001) for physicians, 2.77 (95% CI: 2.15–3.56, *p* < 0.00001) for residents, and 5.05 (95% CI: 1.81–14.13, *p* = 0.002) for nurses (Figure 6). While the numerical OR values suggest a potentially stronger association among nurses, the differences between these population subgroups were not statistically significant (*p* = 0.33).

**Figure 7:**
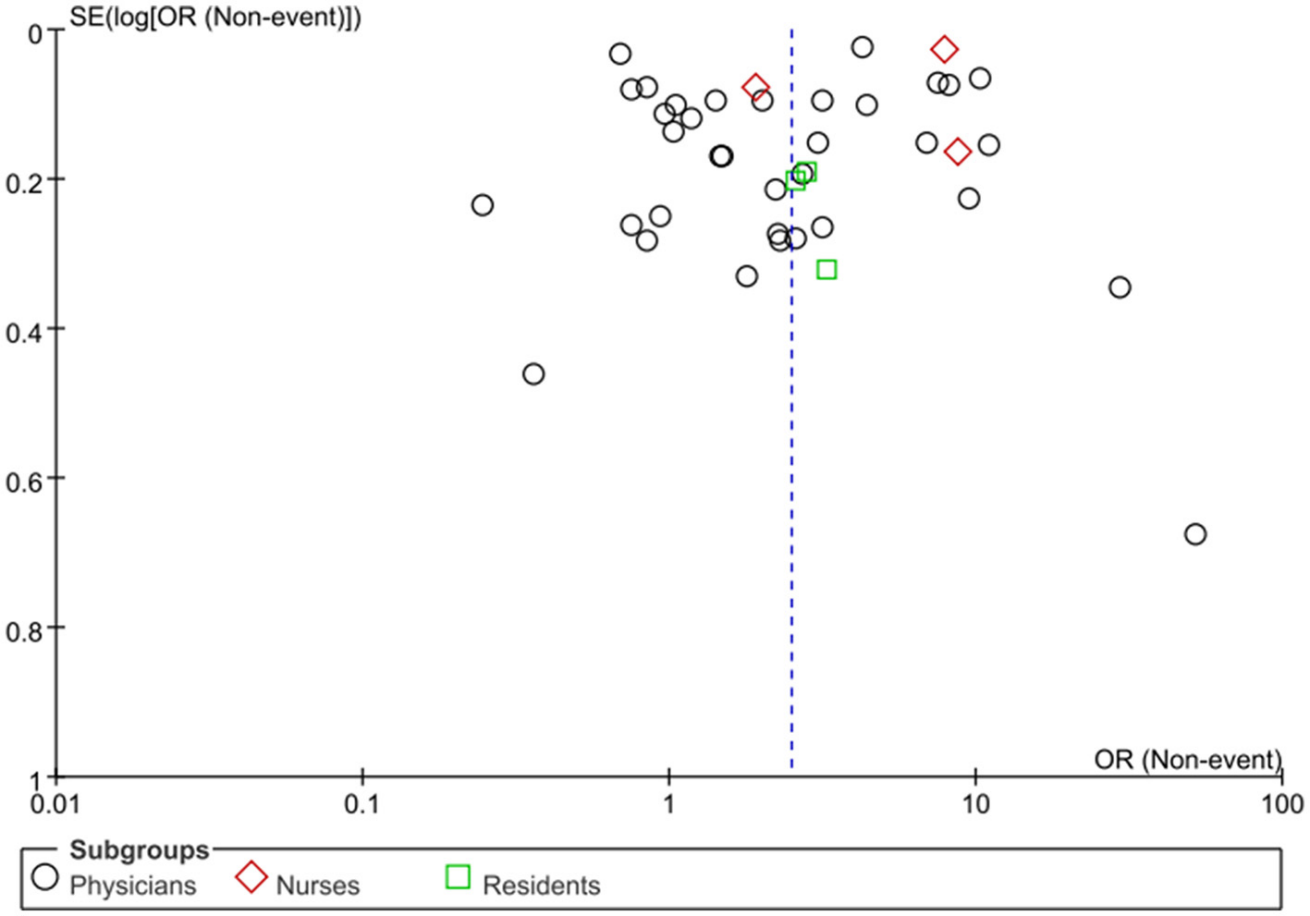
Funnel plot for publication bias.

From a descriptive standpoint, the overall burnout prevalence observed within each population was 38.1% for physicians, 37.5% for residents, and 27.8% for nurses. We emphasize that these prevalence figures represent the absolute burden of burnout within each cohort, whereas the pooled ORs specifically reflect the strength of association between EHR use and burnout risk. A funnel plot assessment suggested no significant evidence of publication bias (Figure 7).

### Main causes of burnout and proposed solutions

We have summarized the factors contributing to burnout among healthcare professionals related to EHR use in Table 2. Poor design and usability of the EHR system were identified as the primary contributing factors. Furthermore, spending excessive time on EHR-related tasks outside of working hours, suboptimal EHR design, redundant alerts, and cumbersome workflows were also identified as key drivers of burnout for healthcare professionals. Among the 41 included studies, 20 specifically mentioned workload factors as a significant exacerbating element of this issue. Additionally, several effective measures to mitigate burnout were proposed. These include optimizing EHR design, increasing the use of scribes to assist with documentation, providing targeted training for clinicians, and implementing mandatory rest periods.

**Table 2:** Risk factors and potentially protective factors associated with EHR-related burnout.

**Author**	**Design**	**Risk factors for burnout**	**Protective factors against burnout**	**Main EHR factors influencing burnout**
Tawfik et al.	Cross-sectional	NICU with ≥10 weekly admissions, nursing care workload, and patient mortality	Burnout recognition education; implementation of burnout interventions at the individual and institutional level	Using EHR outside working or at home; time on using EHR
Shanafelt et al.	Cross-sectional	Using CPOE female gender, emergency medicine, each additional hour per week	Assistant order entry; documentation support	Time spent on clerical tasks
Tawfik et al.	Cross-sectional	HIT frustration, difficulty in falling asleep	Supplemental EHR training; scribes to assist documentation; team-based documentation and inbox management; automating data-entry tasks	Frustrated or stressed by EHR
Kroth et al.	Cross-sectional	Inefficient user interfaces, unpredictable system response times, poor interoperability between systems and excessive data entry	HICT and clinic architectural and process redesign	Proficiency with EHR use; Sufficient time for documentation
Kutney-Lee et al.	Cross-sectional	Employing EHR systems with suboptimal usability	EHR usability	EHR adoption level and teaching status
Olson et al.	Cross-sectional	Poor control over workload, inefficient teamwork, lack of value alignment with leadership, and hectic-chaotic work atmosphere	Improve professional satisfaction; nonphysician order entry	Using EHR outside working or at home; insufficient documentation time
Tai-Seale et al.	Cross-sectional	Female gender and poor control over work schedule	Feeling highly valued; having good control over work schedule; working in a quiet or busy but reasonable environment; assist physician with email work; limit desktop medical work outside working hours (except in emergencies)	Using EHR outside working or at home; number of EHR system-generated in basket messages
Apaydin et al.	Cross-sectional	Managing unscheduled or same-day patients, lack of pharmacist support, administrative work, excessive overall workload, difficulty communicating with other professionals, inadequate care coordination, and answering patient emails	Interventions to facilitate provider-led quality improvement	Managing in-basket messages generated by EHR; responding to EHR alerts
Livaudais et al.	Cross-sectional	Negative perceptions of EHR	Perceiving positive effect of EHR in practice; technical support for EHR when using systems; EHR optimization program	Managing in-basket messages generated by EHR; poor EHR design; dealing with patient-call messages in systems
Tran et al.	Cross-sectional	Clinical full-time equivalents >0.9 and more incomplete messages in inbox	Perception positive attitudes about the effect of EHR or satisfied with EHR	Average additional 10 minutes spent on EHR after each visit; less efficient at completing EHR and inbox information
Marckini et al.	Cross-sectional	Female gender and dissatisfaction for clerical tasks	EHR optimization; improving physician efficiency; and job satisfaction	Managing in-basket messages generated by EHR; dissatisfaction with EHR
Gardner et al.	Cross-sectional	Primary care specialties, female gender, and reporting poor or marginal time for documentation	Perception positive attitudes about the effect of EHR or satisfied with EHR	Excessive data inputting in EHR; using EHR at home; frustrated with EHR
Hilliard et al.	Cross-sectional	High volume of patient call messages in the system and lack of control over workload	Copy and paste used in EHR documentation; assist with inbox tasks and create 2 administrative “desktops”	Using EHR outside working or at home; excessive data inputting in EHR; managing in-basket messages generated by EHR
Higgins et al.	Cross-sectional	Self-compassion, sleep disorder, lacking support from leaders, and poor control over schedules	Peer support, perceived appreciation and meaningfulness in work; maintaining values consistent with practice institution	Poor EHR usability; perception negative attitudes about the effect of EHR
Czernik et al.	Cross-sectional	Frustrated or stressed by EHR	Reducing the burden of documentation tasks; improving EHR usability; interventions to improve the EHR	Poor usability of EHR; information overload; degradation of medical documentation
Melnick et al.	Cross-sectional	lower emotional exhaustion scores, depersonalization scores, and overall rates of burnout	Standardized technical availability	Improving EHR usability
Domaney et al.	Cross-sectional	emotional exhaustion, depersonalization, and low sense of personal accomplishment		The total time spent using EHR per week is 22 hours
Hauer et al.	Cross-sectional	Loss of practicing autonomy, female gender, frustrated with EHR, and increasing insurance and government regulation	Improve the functionality of EHR; enhance physician leadership and involvement; create a center for physician empowerment; create a physician health program	Using EHR outside workday
Gajra et al.	Cross-sectional	Variable reimbursement models, interactions with payers, and increasing treating and caring demands	Use advanced practice providers; hire additional administrative staff; invest in information technology	Excessive data inputting in EHR; frustrated or stressed by EHR; using EHR outside workday
Adler-Milstein et al.	Cross-sectional	Poor self-rated EHR skills	Improve EHR design; scribe or team documentation; reduce documentation requirements	Using EHR outside working or at home; time spent on EHR; system-generated in-basket messages (>114) per week
Somerson et al.	Cross-sectional	Working >80 hours per week, verbal abuse from faculty, educational debt, “scut” work >10 hours per week	Nursing support; duty-hour restrictions; improving EHR functionality and efficiency; adequate, personalized training and support; adequate social work support	Time spent on EHR per week; used EHR >20 hours per week
Melnick et al.	Cross-sectional	Practice location (academic medical center) and medical specialty	Improve EHR usability	Using EHR outside working or at home; poor EHR usability
Coleman et al.	Cross-sectional	Work-related physical pain, work-home conflict, and younger age	Build personal resilience, enhance wellness; peer support; reduce administrative or EHR burden	Using EHR outside working or at home; increased EHR or documentation requirement
Abraham et al.	Cross-sectional	Intraorganizational factors	EHR with multifunctional; reduce high EHR workload; work with supportive colleagues; improve team communication	High EHR workload
Kondrich et al.	Cross-sectional	Feeling undervalued by patients, lacking superior support, little promotion chances, perceived unfair clinical working schedule, and nonacademic environment	Improve physician well-being	Feeling that the EHR detracts from patient care
Kroth et al.	Cross-sectional	Overall stress	Improve EHR design; clinician training; scribes to assist documentation; work at home boundaries; exercise, taking breaks	Information overloading; slow system response; excessive data inputting; fail to navigate quickly; note bloat; patient- clinician relationship interference; fear of missing something; billing oriented notes.
Tajirian et al.	Cross-sectional	Workflow issues	Reduce the administrative burden of EHR; improve EHR	Lower satisfaction and higher frustration with the EHR; poor intuitiveness and usability of EHR
Mandeville et al.	Cross-sectional	HIT-related stress and burnout and emergency medicine	Improved workflow	Daily frustration added by EHR; using EHR outside working or at home
Tiwari et al.	Cross-sectional	Lack of physical exercise and weekly working hours	Teamwork and working satisfaction; self-care training	Poor EHR usability; dissatisfaction with EHR
Sinha et al.	Cross-sectional	Interpersonal disengagement	Lower CLOC ratio (total CLOC time to allocated appointment time); well-established personal resources	Using EHR outside working
Anderson et al.	Cross-sectional	Female gender, younger age, shorter practicing years, and having children at home	Taking 20 days or more of vacation time	Using EHR at home; ≥2-hour patient administration
McPeek-Hinz et al.	Cross-sectional	The gender of the bed doctor	The local work culture	The time spent after work
Nair et al.	Cross-sectional	Working long hours, weekly number of nursing patients, practice environment, disinterested health systems, and dissatisfaction with remuneration	Caring for fewer patients per week	Using EHR outside working or at home; EHR requirements
Jha et al.	Cross-sectional	COVID-19 pandemic and in-house billing	Stay positive; improved EHR design	Documentation through EHR
Esmaeilzadeh and Mirzaei	Cross-sectional	Less direct communication with patients, inadequate training for using HIT, and increasing computerization at work	Positive perceptions of EHR; more policy and legal interventions to ensure meaningful use of EHR	Poor EHR usability; time spent entering data
Holzer et al.	Cross-sectional	Receive COVID-19 patients	Using EHR to streamline clinical care activities; physician task relief	Using EHR outside work; increased EHR workload
Baxter et al.	Cross-sectional	Medical conditions, expletives and/or profanity	NLP analyses of inbasket messages at scale	EHR inbox messages
Wilkie et al.	Cross-sectional	High workload and insufficient resources	Good leadership; prioritize work-life balance	Poor EHR usability
Almulhem et al.	Cross-sectional	Daily work increases the sense of frustration	Further research should be conducted to explore possible solutions	Remote EHR use
Lou et al.	Cross-sectional	The clinical workload of EHR	Mitigate sustained elevations of work responsibilities	Total EHR usage time, patient load, and chart review time
Tajirian et al.	Cross-sectional	Daily frustration	Streamlining prescription processes, enhancing search functionalities, and addressing system inefficiencies	Medication reconciliation and prescription processes; chart navigation and information retrieval; longitudinal medication history; technology infrastructure challenges.

### Sensitivity analysis

**Figure 8:**
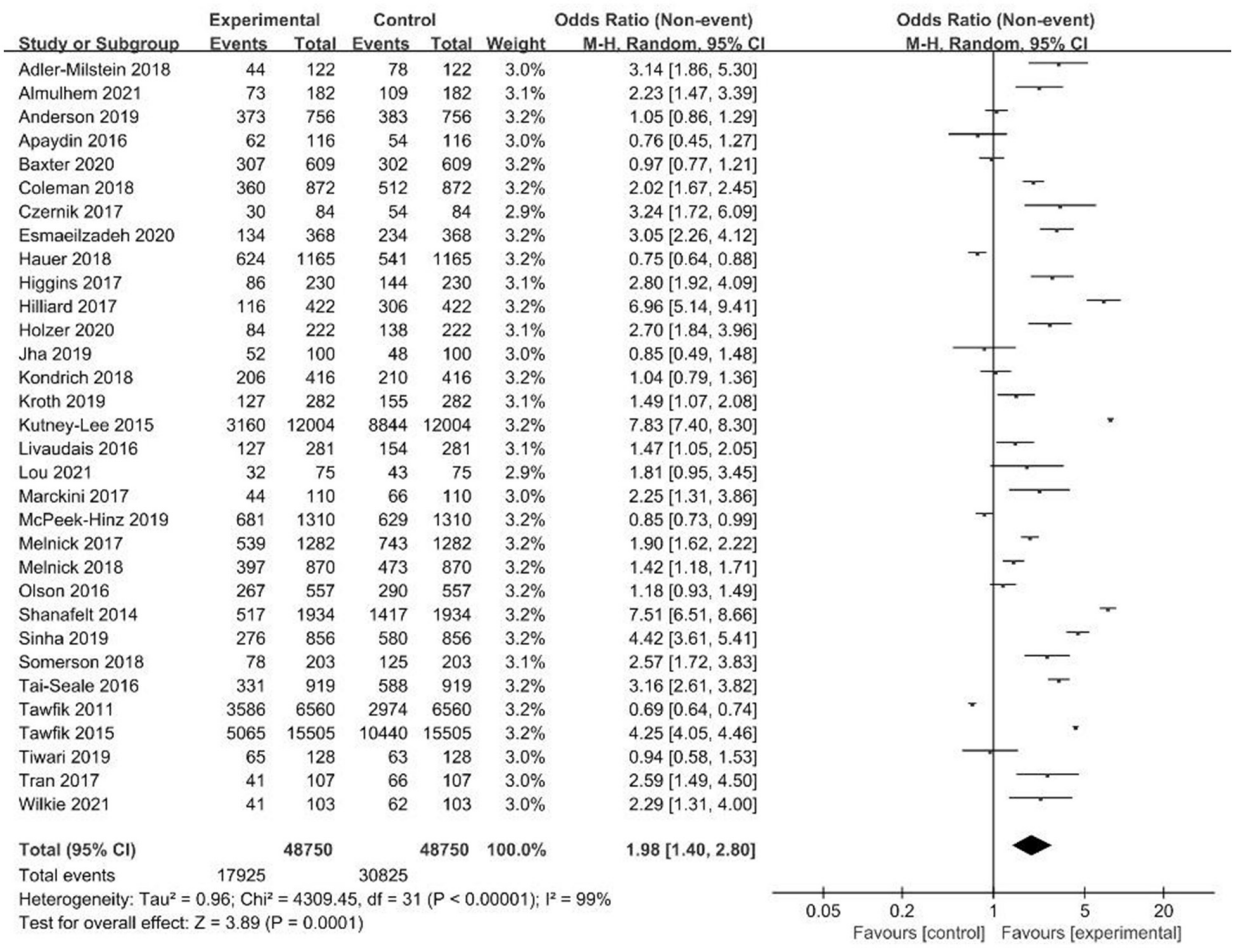
Sensitivity analysis.

**Figure 9:**
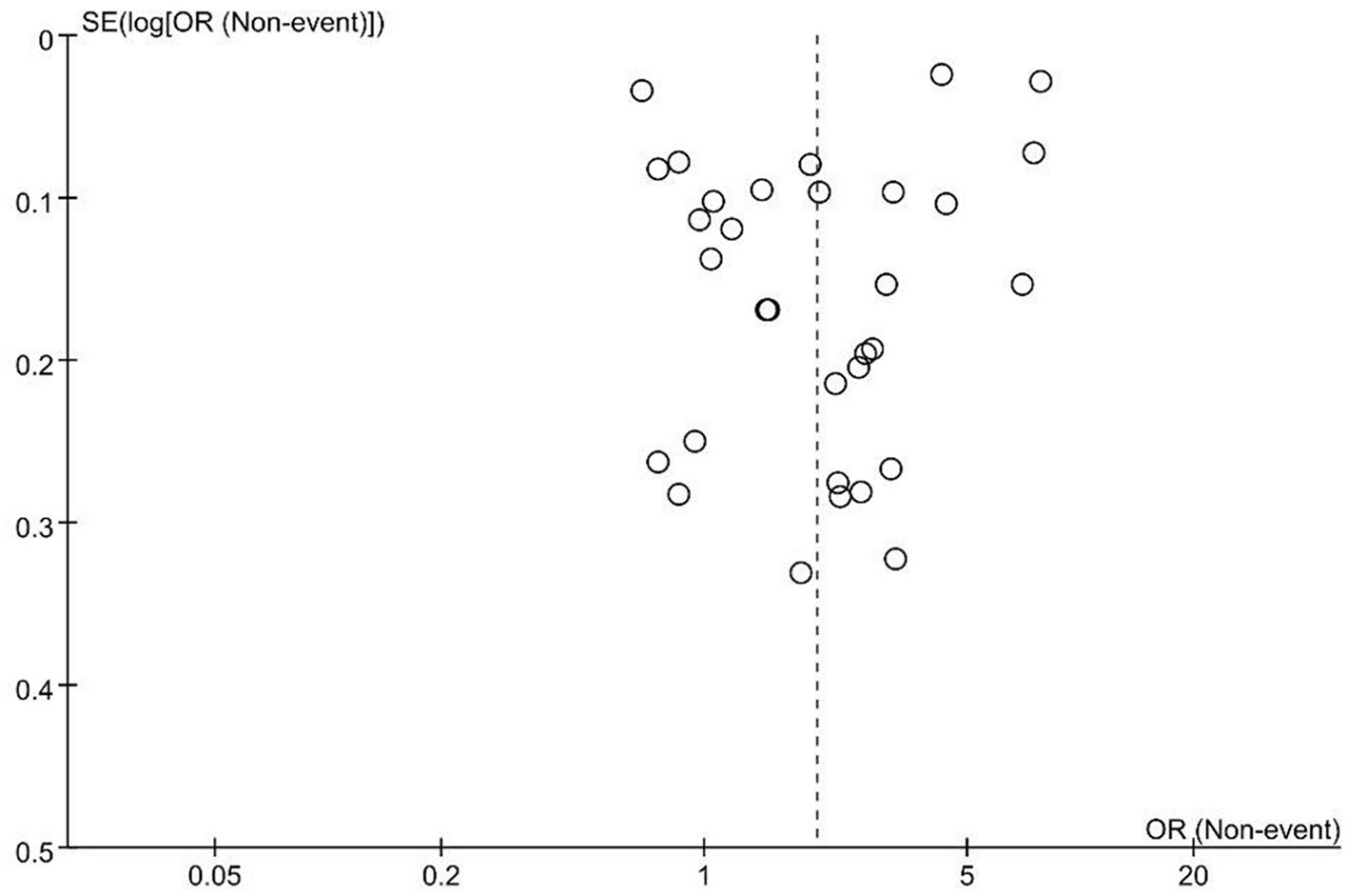
Funnel plot for publication bias.

To ensure the robustness of our findings, a sensitivity analysis was conducted by excluding nine studies identified as outliers, small-scale, or having a high risk of bias. This analysis encompassed 48,750 healthcare professionals, with a descriptive burnout prevalence of 36.8% (17,925/48,750). Despite significant heterogeneity (*I*^2^ = 99%), the random-effects model confirmed that the association between EHR use and increased risk of occupational burnout remained significant (OR = 1.98, 95% CI: 1.40–2.80, *p* < 0.00001) (Figure 8). No significant publication bias was observed (Figure 9). These results demonstrate that the association is stable and not driven by external factors such as study quality or size. The persistent high heterogeneity likely reflects clinical and methodological variations across studies, such as diverse study populations and burnout assessment instruments.

## Discussion

EHR is a comprehensive system for storing patient health information in a digital format. It is designed to be accessible across healthcare institutions and updated in real-time, encompassing the full lifecycle of patient data-including medical history, diagnoses, medications, and laboratory results. The primary goals of EHR implementation are to enhance healthcare quality, optimize service workflows, and promote information coordination. However, poor design and suboptimal application of EHRs can also increase the documentation burden and contribute to burnout among healthcare professionals (4, 17, 18). Burnout is characterized by three core dimensions: emotional exhaustion, depersonalization (or cynicism), and a reduced sense of personal accomplishment (19). This condition not only leads to decreased work efficiency and lower quality care but also places a significant physical and psychological strain on healthcare workers (20, 21). Our meta-analysis reveals a significant association between EHR use and an increased risk of burnout. Furthermore, it uncovers variations in burnout occurrence rates when assessed by different instruments and across different healthcare populations. However, these differences were not statistically significant, suggesting that EHR-related burnout is a pervasive issue affecting multiple groups within the healthcare workforce. Nevertheless, the implementation of targeted, modifiable mitigations tailored to specific healthcare populations has the potential to reduce the incidence of burnout.

MBI-HSS is one of the most widely used instruments for assessing occupational burnout, particularly among healthcare professionals (22). Our subgroup analysis indicated that studies utilizing the MBI-HSS reported a relatively high occurrence rate of burnout. This can likely be attributed to the MBI-HSS's comprehensive, three-dimensional assessment of burnout, which measures emotional exhaustion, depersonalization, and reduced personal accomplishment (22). Each dimension of the scale has well-defined, objective cutoff values, which helps to mitigate the subjective bias inherent in self-reported burnout. In contrast, mini-Z is a brief assessment tool that typically focuses on core burnout symptoms, such as emotional exhaustion and job satisfaction. Due to its limited number of items, it may have lower sensitivity in identifying mild burnout during rapid screening, potentially leading to the relatively lower occurrence rates it reports (23). Other tools included in our analysis comprised non-standard assessment instruments, such as the Stanford Physician Wellness Survey and various custom-designed questionnaires. These tools often have broader assessment scopes and may conflate general work-related fatigue with the specific syndrome of burnout, which could contribute to an inflation of the reported occurrence rates. Although there were numerical differences in the occurrence rates across the three tools, these differences were not statistically significant, indicating that occupational burnout is prevalent regardless of the assessment tool used.

This study confirms through Meta-analysis that the use of EHR is significantly associated with an increased risk of occupational burnout among healthcare workers (OR = 2.49), which was also supported by the results of sensitivity analysis (OR = 1.98). This finding is highly consistent with the results of Wu et al. (13) (OR = 2.43), collectively revealing the robustness of EHR as a driver of occupational burnout. Of particular note is that our analysis, as well as the study by Wu et al., clearly indicates that the time spent on EHR-related tasks outside of working hours is a key and quantifiable risk factor for burnout. Evidence from case-control studies suggests that performing more than 6 h of EHR charting work outside of working hours significantly increases the risk of burnout (24). This “invisible overtime” not only erodes the personal time of healthcare workers, directly leading to emotional exhaustion, but also blurs the boundaries between work and life, serving as one of the core mechanisms triggering occupational burnout.

In terms of the occurrence of EHR-related occupational burnout, the rate among physicians was slightly higher than that among residents and nurses, although the difference across these three groups was not statistically significant. This suggests that the impact of EHRs on burnout is a pervasive issue across multiple healthcare professional groups, with flaws in EHR system design and the significant number of additional hours spent on these systems being common contributing factors. Previous systematic reviews have indicated that research on EHR-related burnout has predominantly focused on physicians (5). As the primary decision-makers in patient care, physicians are required to use EHRs to perform high-frequency and complex documentation tasks, such as recording patient histories, entering orders, and writing progress notes. This demanding and burdensome documentation workload also diminishes physicians' satisfaction with the EHR, a finding consistent with the research of Shanafelt et al. (25). Our review summarizes that a primary cause of burnout among physicians is the excessive time spent on EHRs, which can amount to over 20 additional hours per week and is often completed outside of regular working hours (e.g., at night or on weekends). This form of “invisible overtime” directly exacerbates emotional exhaustion. Furthermore, mandatory, non-essential fields and repetitive alerts within EHR systems can undermine a physician's sense of personal accomplishment, thereby amplifying feelings of burnout. These factors may represent the core reasons for their slightly higher occurrence rate (26). For residents, the need to cope with high-intensity rotating schedules, in addition to spending extra time on EHR-related tasks, is a likely contributor to increased occupational burnout, which aligns with conclusions from prior studies (27, 28). Nurses, in contrast, primarily use EHRs to document the nursing process, a task set that is relatively more standardized and less complex than the comprehensive documentation required of physicians. This may partially account for their lower observed burnout occurrence. However, research indicates that the daily frustrations caused by the EHR and insufficient time for documentation remain key factors contributing to burnout among nurses (9).

This systematic review identifies several potentially modifiable factors that are associated with a reduced risk of EHR-related burnout. However, it is important to note that these strategies are derived primarily from cross-sectional studies and represent observational associations or author recommendations rather than interventions validated by randomized controlled trials (RCTs). Among the proposed measures, the deployment of medical scribes currently holds the most observational support across multiple studies for reducing physician documentation burden. EHR interface optimization and workflow improvements are frequently recommended to enhance efficiency but lack validation through controlled trials. Additionally, while AI/NLP-based documentation assistance shows promise, it remains largely at the expert recommendation stage with emerging pilot data. For physicians, the deployment of dedicated medical scribes or digital documentation solutions has been associated with reduced documentation burden and may be a promising strategy. Furthermore, optimizing the EHR user interface and navigation may enhance workflow efficiency and professional satisfaction (5). For residents, strengthening EHR-specific training has been proposed to lower adaptation barriers and foster a greater sense of mastery and accomplishment, potentially mitigating early-career burnout (29). For nursing staff, developing intuitive, user-friendly nursing documentation modules and streamlining communication workflows have been proposed as potentially beneficial approaches to alleviate daily frustrations caused by EHR interactions (30). Additionally, advancements in artificial intelligence (AI) and natural language processing (NLP) hold promise for automating documentation tasks (31), such as summarizing clinical conversations, drafting clinical notes, and intelligently prioritizing inbox messages (32). However, the application of NLP is not without limitations, such as challenges related to data imbalance (33). Future prospective cohort studies and RCTs are warranted to establish the causal effectiveness and long-term impact of these proposed strategies.

This systematic review has several limitations. First, while the high observed heterogeneity is not uncommon in meta-analyses of occurrence rate, it suggests potential variations in methodologies, definitions of “burnout,” and cultural contexts across the included studies. A random-effects model was used for the pooled analysis in this study because of the extremely high heterogeneity. Although the pooled estimate cannot be interpreted as a single, unified quantitative reference value, it nonetheless demonstrates a clear and statistically meaningful directional trend. In the sensitivity analyses, the direction of the pooled effect size remained consistent, indicating coherence in the overall trend of the study outcomes. Importantly, the high degree of heterogeneity did not alter the direction of the study's main conclusions. Second, the current body of research is predominantly concentrated in North America, which may limit the generalizability of our findings to other healthcare systems. Furthermore, most of the studies were cross-sectional in design, which precludes the establishment of definitive causal relationships. Future research should prioritize prospective cohort studies to better establish causality. There is also a need for greater focus on non-physician groups, particularly nurses and other allied health professionals, as their patterns of EHR interaction and unique sources of stress remain understudied. Finally, evaluating the effectiveness of various targeted mitigations for these specific populations represents a critical next step for the field. In the future, we will undertake actual research in this direction.

## Conclusion

This systematic review and meta-analysis demonstrate that EHR use is significantly associated with an increased risk of occupational burnout across multiple healthcare professional groups, including physicians, nurses, and residents. Primary contributing factors identified include poor EHR design, excessive documentation time demands, and heavy administrative burdens. Targeted mitigation strategies, such as EHR system optimization and the deployment of medical scribes, show potential in reducing burnout among physicians. For nursing staff, developing intuitive documentation modules and streamlining communication workflows may help alleviate occupational stress. These findings provide a critical evidence base for healthcare institutions to optimize EHR implementation and develop tailored interventions. Future prospective studies and RCTs are essential to validate the causal effectiveness of these proposed mitigation approaches.

## Data Availability

The original contributions presented in the study are included in the article/Supplementary material, further inquiries can be directed to the corresponding author.
