# Supplementary material for: Electronic health records-related determinants of healthcare professionals' burnout and mitigation strategies: systematic review and meta-analysis
Source: Front Public Health. 2026 Mar 13;14:1751521. doi: 10.3389/fpubh.2026.1751521 (PMC13021678; doi:10.3389/fpubh.2026.1751521)
Supplement: Supplementary file 1 [file supplementary_file_1.docx]

Search strategy for Pubmed on July 31, 2025

Records identified: 451 filter: published between 2005/1/1-2025/07/31 and in English

((((((burnout[Title/Abstract]) OR (burn-out[Title/Abstract])) OR (alert fatigue[Title/Abstract])) OR (alarm fatigue[Title/Abstract])) OR (exhaustion[Title/Abstract])) AND (((((((physicians[Title/Abstract]) OR (doctors[Title/Abstract])) OR (medical staff[Title/Abstract])) OR (healthcare professional[Title/Abstract])) OR (clinicians[Title/Abstract])) OR (nurses[Title/Abstract])) OR (medical student[Title/Abstract]))) AND ((((((((electronic health record[Title/Abstract]) OR (EHR[Title/Abstract])) OR (electronic medical record[Title/Abstract])) OR (EMR[Title/Abstract])) OR (computerized physician order entry[Title/Abstract])) OR (CPOE[Title/Abstract])) OR (clinical decision support system[Title/Abstract])) OR (CDSS[Title/Abstract]))

Search strategy for Embase on 31 July 2025

Records identified: 699 filter: published between 2005/1/1-2025/07/31 and in English

('electronic health record':ti,ab,kw OR ehr:ti,ab,kw OR 'electronic medical record':ti,ab,kw OR emr:ti,ab,kw OR cpoe:ti,ab,kw OR 'computerized physician order entry':ti,ab,kw OR cdss:ti,ab,kw OR 'clinical decision support system':ti,ab,kw) AND (physicians:ti,ab,kw OR doctors:ti,ab,kw OR nurses:ti,ab,kw OR clinicians:ti,ab,kw OR 'medical staff':ti,ab,kw OR 'healthcare professional':ti,ab,kw OR 'medical student':ti,ab,kw) AND (burnout:ti,ab,kw OR 'exp burnout/ rr burn-out':ti,ab,kw OR 'burn out':ti,ab,kw OR exhaustion:ti,ab,kw OR 'alert fatigue':ti,ab,kw OR 'alarm fatigue':ti,ab,kw)

Search strategy for Web of Science on 31 July 2025

Records identified: 1456 filter: published between 2005/1/1-2025/07/31 and in English

Physician (Topic) OR doctor (Topic) OR clinician (Topic) OR nurses (Topic) OR medical staff (Topic) OR healthcare professional (Topic) OR medical student (Topic) AND electronic health record (Topic) OR EHR (Topic) OR electronic medical record (Topic) OR EMR (Topic) OR CPOE (Topic) OR computerized physician order entry (Topic) OR CDSS (Topic) OR clinical decision support system (Topic) AND burnout (Topic) OR burn-out (Topic) OR burn out (Topic) OR exhaustion (Topic) OR alert fatigue (Topic) OR alarm fatigue (Topic)
