# Supplementary material for: Electronic health records-related determinants of healthcare professionals' burnout and mitigation strategies: systematic review and meta-analysis
Source: Front Public Health. 2026 Mar 13;14:1751521. doi: 10.3389/fpubh.2026.1751521 (PMC13021678; doi:10.3389/fpubh.2026.1751521)
Supplement: Supplementary file 3 [file supplementary_file_3.docx]

**Supplementary table 3** Joanna Briggs Institute checklist for the cross-sectional studies included.

| Title | Author | 1 | 2 | 3 | 4 | 5 | 6 | 7 |
| --- | --- | --- | --- | --- | --- | --- | --- | --- |
| Relationship between clerical burden and characteristics of the electronic environment with physician burnout and professional satisfaction | Shanafelt et al | No | Moderete | Moderete | Moderete | Yes | Yes | Yes |
| Factors associated with provider burnout in the NICU | Tawfik et al | No | No | Yes | No | Yes | Moderete | Moderete |
| Frustration with technology and its relation to emotional exhaustion among health care workers: cross-sectional observational study | Tawfik et al | Moderete | Yes | Moderete | Moderete | Yes | Yes | Yes |
| The electronic elephant in the room: Physicians and the electronic health record | Kroth et al | Yes | Yes | Yes | Moderete | Moderete | Yes | Moderete |
| Electronic Health Record Usability: Associations With Nurse and Patient Outcomes in Hospitals | Kutney-Lee et al | Moderete | Yes | Yes | No | Moderete | No | Moderete |
| Cross-sectional survey of workplace stressors associated with physician burnout measured by the Mini-Z and the maslach burnout inventory | Olson et al | Yes | Moderete | Yes | No | Yes | Yes | Moderete |
| Physicians' well-being linked to in-basket messages generated by algorithms in electronic health records | Tai-Seale et al | Yes | Moderete | Moderete | Moderete | Yes | Moderete | Moderete |
| Association between difficulty with VA patient-centered medical home model components and provider emotional exhaustion and intent to remain in practice | Apaydin et al | No | No | Yes | Yes | Yes | Moderete | Moderete |
| Perceived value of the electronic health record and its association with physician burnout | Livaudais et al | No | Moderete | Yes | Moderete | Yes | Yes | Yes |
| Burnout and EHR use among academic primary care physicians with varied clinical workloads | Tran et al | No | Moderete | Yes | Moderete | Yes | Moderete | Yes |
| Electronic health record associated stress: a survey study of adult congenital heart disease specialists | Marckini et al | Moderete | No | Moderete | Moderete | Yes | Yes | Yes |
| Physician stress and burnout: the impact of health information technology | Gardner et al | Moderete | No | Yes | Moderete | Yes | Yes | Moderete |
| Are specific elements of electronic health record use associated with clinician burnout more than others | Hilliard et al | Moderete | No | Yes | Moderete | Moderete | Yes | Yes |
| Burnout, professional fulfillment, intention to leave, and sleep-related impairment among radiology trainees across the United States (US): a multisite epidemiologic study | Higgins et al | Moderete | No | Yes | Moderete | Yes | Yes | Yes |
| Hospitalist perceptions of electronic health records: a multi-site survey | Czernik et al | No | Moderete | Moderete | Moderete | Yes | Moderete | Moderete |
| The association between perceived electronic health record usability and professional burnout among US nurses | Melnick et al | Yes | Moderete | Yes | Moderete | Yes | Moderete | Yes |
| Exploring the Association Between Electronic Health Record Use and Burnout Among Psychiatry Residents and Faculty: a Pilot Survey Study | Domaney et al | Moderete | Yes | Moderete | No | Yes | Yes | No |
| Physician burnout in Wisconsin: an alarming trend affecting physician wellness | Hauer et al | Moderete | Moderete | Moderete | No | Yes | Yes | Moderete |
| Frequency and causes of burnout in US community oncologists in the era of electronic health records | Gajra et al | No | No | Moderete | No | Yes | Moderete | Yes |
| Electronic health records and burnout: time spent on the electronic health record after hours and message volume associated with exhaustion but not with cynicism among primary care clinicians. | Adler-Milstein et al | Moderete | No | Moderete | Yes | Moderete | Yes | Yes |
| Burnout among United States orthopaedic surgery residents | Somerson et al | Moderete | Yes | Yes | No | Yes | Moderete | Yes |
| The association between perceived electronic health record usability and professional burnout among US physicians | Melnick et al | Yes | Moderete | Yes | Moderete | Yes | Moderete | Yes |
| Vascular surgeon wellness and burnout: a report from the Society for Vascular Surgery Wellness Task Force | Coleman et al | Moderete | No | Yes | Moderete | Yes | Moderete | Yes |
| Use of multifunctional electronic health records and burnout among primary care nurse practitioners | Abraham et al | Moderete | No | Yes | Moderete | Moderete | Moderete | Yes |
| Burnout in pediatric emergency medicine physicians: a predictive model | Kondrich et al | Yes | Moderete | Yes | No | Yes | Yes | Yes |
| Association of electronic health record design and use factors with clinician stress and burnout | Kroth et al | Moderete | Moderete | Moderete | Moderete | Yes | Yes | Yes |
| The influence of electronic health record use on physician burnout: cross-sectional survey | Tajirian et al | No | No | Yes | No | Yes | Moderete | Moderete |
| Use of health information technology by Rhode Island physicians and advanced practice providers | Mandeville et al | Moderete | Moderete | Yes | No | Yes | No | Moderete |
| High burden of burnout on rheumatology practitioners | Tiwari et al | No | No | Yes | No | Moderete | Moderete | Yes |
| Novel nonproprietary measures of ambulatory electronic health record use associated with physician work exhaustion. Appl Clin Inform | Sinha et al | No | Moderete | Yes | No | Yes | Moderete | Moderete |
| Burnout among US gastroenterologists and fellows in training: identifying contributing factors and offering solutions | Anderson et al | Moderete | Moderete | No | No | Yes | Moderete | Yes |
| Clinician Burnout Associated With Sex, Clinician Type, Work Culture, and Use of Electronic Health Records | McPeek-Hinz et al | Moderete | Moderete | Yes | Yes | No | Moderete | Yes |
| Burnout among nephrologists in the United States: a survey study | Nair et al | Moderete | Yes | Yes | Moderete | Yes | Yes | Yes |
| The effect of COVID-19 on interventional pain management practices: a physician burnout survey | Jha et al | No | No | No | No | Yes | No | Yes |
| Using electronic health records to mitigate workplace burnout among clinicians during the COVID-19 pandemic: field study in Iran | Esmaeilzadeh and Mirzaei | Moderete | Moderete | Yes | Moderete | Yes | Yes | Yes |
| Impact of changes in EHR use during COVID-19 on physician trainee mental health | Holzer et al | Moderete | No | Yes | No | Yes | Yes | Moderete |
| Association of Electronic Health Record Inbasket Message Characteristics With Physician Burnout | Baxter et al | Yes | Moderete | Moderete | Yes | Yes | Yes | Moderete |
| Evolution of a physician wellness, engagement and excellence strategy: lessons learnt in a mental health setting | Wilkie et al | Yes | No | Yes | No | Yes | Yes | Moderete |
| Stress and Burnout Related to Electronic Health Record Use among Healthcare Providers during the COVID-19 Pandemic in Saudi Arabia: A Preliminary National Randomized Survey | Almulhem et al | Yes | No | Moderete | Moderete | Moderete | Yes | Yes |
| Temporal Associations Between EHR-Derived Workload, Burnout, and Errors: a Prospective Cohort Study | Lou et al | Yes | No | Moderete | Yes | Moderete | Yes | Moderete |
| Assessing the Impact on Electronic Health Record Burden After Five Years of Physician Engagement in a Canadian Mental Health Organization: Mixed-Methods Study | Tajirian et al | Moderete | Moderete | Yes | No | Yes | No | Moderete |
